# A Perspective on Adventitious Root Formation in Tree Species

**DOI:** 10.3390/plants9121789

**Published:** 2020-12-17

**Authors:** Carmen Díaz-Sala

**Affiliations:** Department of Life Sciences, University of Alcalá, 28805 Alcalá de Henares, Madrid, Spain; carmen.diazsala@uah.es; Tel.: +34-918-855-051

**Keywords:** adventitious rooting, competence, developmental reprogramming, recalcitrance

## Abstract

Adventitious root formation is an organogenic process, regulated at several levels, that is crucial for the successful vegetative propagation of numerous plants. In many tree species, recalcitrance to adventitious root formation is a major limitation in the clonal propagation of elite germplasms. Information on the mechanisms underlying the competence for adventitious root formation is still limited. Therefore, increasing our understanding of the mechanisms that enable differentiated somatic cells to switch their fates and develop into root meristematic cells, especially those involved in cell developmental aging and maturation, is a priority in adventitious root-related research. The dynamic cell wall–cytoskeleton, along with soluble factors, such as cellular signals or transcriptional regulators, may be involved in adult cell responses to intrinsic or extrinsic factors, resulting in maintenance, induction of root meristematic cell formation, or entrance into another differentiating pathway.

## 1. Introduction

Adventitious root formation is an organogenic process regulated at several levels that is crucial for the successful vegetative propagation of numerous plant species. The process is useful in forestry and horticulture for the clonal propagation and conservation of elite germplasms [1]. In many tree species, especially those of forest trees, recalcitrance to adventitious root formation, especially during the maturation stage, is a major limitation for the clonal propagation of elite germplasms [2,3,4,5]. Information on the mechanisms underlying the competence for adventitious root formation is still limited. Thus, understanding the mechanisms that enable differentiated somatic cells to alter their fates and develop into root meristematic cells, especially those related to the organism’s developmental age and maturation, is a priority in adventitious root-related research [6,7]. Several explanations regarding the capacity of cell reprogramming to induce adventitious root meristem have been proposed, and it appears that several factors and mechanisms, perhaps operating independently, are involved [1,6,7,8,9]. The dynamic switching of cell fate during adventitious root formation results from regulatory interactions at various levels. The effects of spatio-temporal cellular and tissue signals, the crosstalk between hormones and key transcriptional regulators, and a dynamic cascade of regulatory changes in gene expression involved in organ patterning, regulate the cell fate-related modifications that enable a differentiated somatic cell to reactivate programs that lead to the induction of adventitious root meristem formation [6,7,8,9].

Transcriptome and proteome analyses of adventitious root formation have been recently performed in easy- and difficult-to-root tree species in response to auxin and other factors, such as hydrogen peroxide or etiolation [10,11,12,13,14,15,16]. Evidence of wound and stress responses, plant hormonal metabolism, carbohydrate and energy metabolism, and cell cycle and cellular dynamics, as well as transcriptional regulation, has been documented during adventitious root formation in several tree species. Despite increasing knowledge of the molecular basis of adventitious rooting, how these signaling pathways are translated into changes in cell behavior, and how cell identity changes affect developmental signaling, resulting in a rechanneling of the developmental memory, remain poorly understood [6,9]. Because morphogenesis involves dynamic cellular processes, tracking real-time changes in cell dynamics will be crucial for further understanding these processes [6,9].

Most tree species share a common trait in adventitious root induction, the absolute requirement of exogenous auxin treatments [2,3,4,5]. Auxin accumulation at the rooting sites is an initial response of rooting-competent tissues that is lacking in non-competent tissues of pine [17,18]. The induction of the adventitious rooting program depends on the presence of exogenous auxin and on the degree of auxin localization at the rooting cells after excision in rooting-competent cuttings of pine, and this process is antagonistic to the induction of the xylogenesis program [6,17,18,19,20]. Pizarro and Díaz-Sala [20] demonstrated that xylem parenchymal and procambial cells located at the xylem poles of rooting-competent pine hypocotyl cuttings may follow different morphogenic pathways, including adventitious root meristem or cambium differentiation and xylem formation, depending on the presence of exogenous auxin and the directional auxin flow. The disruption of auxin accumulation at the rooting sites in the presence of polar auxin transport inhibitors induces xylem formation and inhibits rooting [17,20]. Gibberellins may act within this pathway by promoting the induction of cambial proliferation and xylem formation and by inhibiting rooting [20]. The low rooting capacity of green branches, compared with dark-grown branches and the branches that underwent short de-etiolation, in avocado is also associated with increases in cambium activity and xylem formation in the former [21]. Immanen et al. [22] showed that auxin and cytokinin form distinct and partially overlapping distribution domains across the vascular cambium of poplar. Gibberellin reaches its maximum level in developing xylem cells and may act with auxin to modulate the cambial activities required for the transition to the maturation phase. Johnsson et al. [23] described an interplay between auxin and gibberellin required to initiate fiber differentiation. An interference in auxin transport leads to premature secondary cell-wall deposition and fiber-bundle formation. Furthermore, Ilegems et al. [24] showed that the effects of specific transcription factors on the canalization of auxin flow are essential for cell division and cell alignment during procambial cell formation in *Arabidopsis*. Pizarro and Díaz-Sala [6,20] speculated that modifications in the auxin flow at the rooting sites and the priming of cambial cells to differentiate into xylem during tree ageing are associated with the maturation-related decline of adventitious root formation. The priming of cambial cells to induce xylogenesis may mark a point of no return after which adult cells cannot induce adventitious root meristem formation [6,20].

In animal systems, dynamic extracellular matrix-modulated signaling plays important roles in remodeling morphogenesis by regulating stem-cell retention and lineage commitment. The extracellular matrix, along with soluble factors secreted by cells present within the niche, help regulate the decision between stem cell self-renewal and differentiation [25]. Specific and dynamic changes in the interactions between the cell wall and cytoskeleton, affecting polarization of auxin efflux carriers in rooting progenitor cells prior to and after the point of no return, when an irreversible decline in adventitious root formation occurs, may represent possible targets for the developmental, environmental, hormonal, and epigenetic regulation of the maturation-related decline in adventitious root formation [6,9,20].

## 2. Cell Dynamic Components as Targets of Competence for Adventitious Rooting

Signaling modulated by a dynamic cell wall–cytoskeleton plays important roles in lateral organ formation [26]. Evidence from model plants also suggests that cell wall–cytoskeleton dynamics have important functions in adventitious root formation [26]. However, knowledge of the effects of the cell wall–cytoskeleton dynamics on the recalcitrance for adventitious root formation is still limited [6,9,20,26].

Molecular regulatory studies have indicated roles for cytoskeletal and cell-wall modifications in the maturation-related decline of adventitious root formation in pine [27,28]. More recently, Rigal et al. [29] found that genes involved in cell-wall remodeling, such as glycoside hydrolases, pectate lyases, pectin esterases, and expansins, have high transcript levels during the early stage of adventitious root formation in poplar.

Eliyahu et al. [30] found that the high rate of callus formation in response to auxin in cuttings from mature compared with juvenile trees, which form roots, resulted from differences in cell-wall properties in *Eucalyptus brachyphylla*. In roots, methylesterified pectin was localized at the cell junctions; however, in the callus parenchyma-like cells, it was localized throughout the cell wall. Furthermore, callus cells showed high levels of demethylesterified pectin compared with root cells. The required synthesis of an appropriate cellulose for the induction of adventitious root and callus formation confirmed the involvement of cell-wall organization and modification in adventitious rooting. In apple rootstocks, the levels of tubulin α-3 increased while those of an α-tubulin suppressor decreased in indol-3-butyric acid (IBA)-treated samples during adventitious rooting [31]. Furthermore, several proteins related to cell-wall properties, including xyloglucan endotransglucosylase/hydrolase proteins, expansin-like protein, pectin lyase-like superfamily proteins, cellulase, and an α-l-arabinofuranosidase, showed significantly greater fold changes in IBA-treated samples of competent cuttings. These data suggest the involvement of not only cell-wall modifications, but also microtubule dynamics during the induction phase of adventitious rooting. The cytoskeleton has also been associated with the maturation-related decline of adventitious root formation in *Eucalyptus grandis*. The developmental and auxin-dependent regulation of genes coding for several microtubule and microtubule-associated proteins in juvenile and adult cuttings of this species have been described [32]. Treatments with microtubule-disrupting drugs increase the rooting capacity of adult cuttings, suggesting correlations between adventitious rooting and microtubule remodeling and their relevance to the maturation-related decline in adventitious root formation [32].

Recently, Duman et al. [21] demonstrated that short-term de-etiolation increases the rooting capacity in avocado rootstock branches compared with green branches, which show increases in cambium activity and xylem formation. The enriched expression of xyloglucan endotransglucosylases/hydrolases and relatively low expression levels of pectin methylesterases and polygalacturonases compared with in green branches may be related to adventitious root formation in avocado. Reductions in xyloglucan staining in the cambium and parenchyma of white branches, in demethylesterified pectin in the cambium of green branches, and in methylesterified pectin in the parenchyma of green branches are associated with changes in the expression levels of specific cell-wall genes.

Xylogenesis is a polarized cell-differentiation process that also involves specific interactions between the cell walls and cytoskeletons of progenitor cells. Xylemdifferentiation involves the molecular mechanisms that underlie dynamic cell-wall formation, which is determined by cortical microtubules and plant microtubule-associated proteins [33,34,35]. Furthermore, master transcription factors involved in xylem differentiation regulate cell-wall formation during xylogenesis [36].

Endogenous or environmental regulators may successfully modify the cell-maturation and -differentiation processes toward adventitious root formation, depending on the flexibility of the cell wall and the cytoskeleton interactions at specific adult cambial cell developmental stages, before commitment to xylem differentiation, resulting in a new cell polarity and the organization of a root meristem [6,9,20].

## 3. Transcriptional Regulation of Adventitious Root Formation

Transcription factors are the main players in regulatory modules controlling auxin gradients, positional information, and polarity field development, resulting in a cross-regulatory network that is involved in organ formation [37]. Several groups of transcription factors regulate key developmental processes in adventitious root formation, such as plant growth regulator-responsive transcription factors or transcription factors from WRKY, NAM/ATAF/CUC (NAC), APETALA2 (AP2), HOMEODOMAIN-LEUCINE ZIPPER (HD-ZIP I), GIBBERELLIC-ACID INSENSITIVE, REPRESSOR of GAI and SCARECROW (GRAS) and WUSCHEL, as well as WUSCHEL-related HOMEOBOX families [10,11,13,15,16,29,38,39,40,41,42]. The expression of genes coding for transcription factors involved in stem-cell identity is a common feature during the induction of somatic embryogenesis and organogenesis [9], and the induction of transcription factors related to meristematic programs may be associated with the capacity for adventitious rooting in tree species [43].

The WUSCHEL, WUSCHEL-related, and GRAS families of transcription factors are involved in the specific maintenance of stem cells in both primary and secondary plant meristems, as well as in early root meristem and root radial patterning and maintenance, in *Arabidopsis* [44,45]. The expression of homologs of these families has been described during adventitious rooting in tree species [41,42]. The overexpression of WUSCHEL-related homeobox PeWOX11a and PeWOX11b from *Populus euphratica* results in an increased number of adventitious roots. Furthermore, the numbers of induced ectopic roots in the aerial parts significantly increased [46]. The WUSCHEL PtoWUSa and WUSCHEL-related homeobox 5a, PtoWOX5a, are involved in adventitious root induction and development in *Populus tomentosa* [47,48]. In addition, PtoWUSa and PtoWOX5a-overexpressing plants have modified expression levels of genes involved in auxin- and cytokinin-signaling pathways and polar auxin transport, specifically the efflux transport of auxin, as well as those involved in the cell cycle and root development. In the conifer *Larix kaempferi*, WUSCHEL-related homeobox 4, LkWOX4, is expressed in immature xylem, phloem, and roots, and it is involved in adventitious root formation. An analysis of LkWOX4-overexpressing poplar plants indicates that LkWOX4 may be involved in the adventitious root initiation and development, interacting with auxin-signaling pathways, root meristem identity-related transcription factors, and root development-related genes [49]. In addition, two *PpWOX13*-like genes are involved in the control of cell-wall loosening, which facilitates stem-cell formation, in *Physcomitrella patens* [50]. Roles in activating or inducing stem cell niches, which develop into adventitious roots, by interacting with auxin and other signaling pathways, as well as with pathways involved in cell-wall modification, have been proposed for these genes [46,47,48,49,50].

The expression levels of the GRAS’ SCARECROW and SHORT-ROOT homologs have been associated with adventitious rooting in poplar, *Taxodium* hybrids, sugi, walnut, chestnut, and pine [11,15,16,17,41,51,52,53,54,55]. Furthermore, the expression of a specific subset of GRAS family transcription factors is associated with the maturation-related decline of adventitious root formation in both pine and chestnut [17,52,53,54,55]. A subset of other genes is also expressed in an auxin-, age-, or development-dependent manner during adventitious root formation in pine, before the onset of cell division that leads to root meristem formation [17]. The individual genes within each *GRAS* group may have acquired different and specialized functions, some of which may be related to the competence and reprogramming of adult cells to form adventitious roots. In addition, the asymmetric increase in mRNA during the earliest stages of adventitious root formation in the cambial region and in rooting-competent cells that were not detected in non-competent cuttings, which had an mRNA diffusing signal, suggests the presence of specific cellular signaling pathways or specific factors in pine and chestnut. These may be distributed in cell type- and developmental stage-specific contexts in the tissues involved in rooting, which could be crucial for rooting capacity [17,53,54,55]. Similarly, using laser capture microdissection in both rooting-competent juvenile and non-competent mature cuttings of black walnut, Stevens et al. [52] showed that the site-specific transcriptional activation of *SCARECROW LIKE-1*, as well as *AUXIN RESPONSE FACTORS* and *SHORT-ROOT*, is associated with adventitious root formation [52]. The greatest transcript abundance in rooting-competent cuttings was restricted to root progenitor cells, while recalcitrant cuttings had diffuse mRNA signals among tissue types. These findings support the conclusion that the recalcitrance to adventitious root development results from the failure of rooting-competent cells to perceive cellular or molecular signals initiated by auxin [2,3,4,5], and they indicate the evolutionary conservation of this response in distantly-related forest tree species [17,52,53,54,55]. The asymmetric auxin distribution detected in rooting-competent tissues of pine after excision [17] matched the locations where specific *GRAS* genes were expressed [17,53,54,55]. The differential expression of *GRAS* genes and their varied responses to exogenous auxin in rooting-competent and non-competent cuttings [17,53,54,55] during adventitious rooting may indicate the local involvement of specific GRAS transcription factors in rooting, by affecting the auxin distribution, the division of certain cell types, or other mechanisms. The overlap in the temporal and spatial distributions of auxin and *GRAS* genes [17,53,54,55] may indicate crosstalk between the signaling pathways, perhaps establishing response domains that activate a cascade of other *GRAS* genes or root-determining factors before the resumption of cell division.

## 4. Conclusions and Perspectives

Age- and maturation-related trends result from complex interactions among extrinsic and intrinsic factors [4]. The mechanisms that cause a cell to maintain or lose its developmental competence are unknown. Cues within the meristem and/or developing or differentiating cells involve differential gene expression and epigenetic changes during maturation that regulate the genes needed for the expression of specific maturation-related traits. Antagonism between adventitious root formation and xylogenesis could then involve the expression of an array of signaling genes and a functionally complex network of molecular regulations that change during maturation to favor xylem differentiation with the loss of cell reprogramming into a root primordium [6,20]. Furthermore, cell-wall mechanical properties also determine the highly localized regulation of cell dynamics by cell wall-mediated signaling, cell wall-induced alterations in tissue structure, and the regulation of tissue differentiation by chemical and physical properties of the cell wall. A major future challenge will be to integrate the multiple roles of cell wall–cytoskeleton interactions towards xylogenesis or adventitious root formation with data on the many critical soluble regulators, such as cellular signals or transcriptional regulators, involved in adventitious root formation to provide a more complete understanding of recalcitrance.
